# The Aetiology of Sensory Impairment in Pelvic Organ Prolapse: A Prospective Cohort Study

**DOI:** 10.1007/s00192-025-06412-7

**Published:** 2025-12-02

**Authors:** Charlotte Mahoney, Jenny Myers, Anthony Smith, Fiona Reid

**Affiliations:** 1 The Warrell Unit, St Mary’s Hospital, Manchester University Hospitals NHS Foundation Trust, Manchester Academic Health Science Centre, Oxford Road, Manchester, M13 9WL UK; 2 Institute of Human Development, Faculty of Medical & Human Sciences, University of Manchester, Manchester, UK

**Keywords:** Aα and Aβ sensory nerve fibres, Pelvic organ prolapse, Perception thresholds, Postnatal, Quantitative sensory testing, Vibration and stretch sensation

## Abstract

**Introduction and Hypothesis:**

Women with pelvic organ prolapse (POP) have reduced vaginal sensation. Vaginal birth has been associated with both sensory nerve injury and prolapse. It is unclear whether the impaired vaginal sensation seen in POP is caused during childbirth or by the prolapsing tissues themselves.

**Methods:**

The objective was to perform to our knowledge the first study to compare vaginal sensation between women with postnatal POP and women without postnatal POP. A prospective cohort study (Canadian task force classification II-2) of 124 primiparous women who underwent Pelvic Organ Prolapse Quantification examination and neurophysiology at 3 and 6 months postnatally was carried out. Women underwent vibration quantitative sensory testing for Aβ nerve function and stretch quantitative sensory testing for Aα nerve function. POP was defined a priori as the distal-most portion of the vaginal wall at or below the hymen.

**Results:**

There was no difference in age or BMI between women with POP and women without POP. Vaginal vibration and stretch sensation were reduced in women with POP compared with controls at 3 months postnatally (*p* = 0.021 and 0.007 respectively). At 6 months postnatally vibration sensation remained impaired in the POP group, whereas stretch sensation had recovered compared with the control group (*p* < 0.001 and 0.498 respectively).

**Conclusions:**

Vaginal sensation was reduced in women with postnatal POP suggesting that childbirth might contribute to the impaired vaginal sensation seen in women with POP in later life. Further work is required to determine if the impairment persists and is the reason for impaired sensory function seen in women with POP in later life.

## Introduction

Pelvic organ prolapse (POP) affects 1 in 10 women worldwide and causes a significant public health burden [1–3]. Despite this the causes of POP remain incompletely understood, but involve injury to connective tissues, muscles, and motor and sensory motor nerves during vaginal birth [4–6].

Women with POP demonstrate reduced vaginal sensation compared with healthy controls irrespective of age, suggesting that age-related nerve degeneration might not be a factor [7, 8]. Vaginal birth has been shown to cause both pudendal sensory nerve injury and prolapse [4, 9, 10]. It is unclear whether the impaired vaginal sensation seen in POP is caused during childbirth or by the prolapsing tissues themselves.

Neurophysiology assessment of vaginal sensation can be performed using quantitative sensory testing (QST) whereby the minimum stimulus for a woman to perceive a sensation is measured, called the perception threshold [11]. QST provides a reliable assessment of the entire sensory pathway using a validated protocol. Different stimuli can be used to assess different nerve fibres, with Aα sensory nerve function measured using stretch sensation and Aβ nerves assessed using vibration sensation [12, 13].

We hypothesised that if the reduced vaginal sensation seen in women with POP was caused by vaginal birth it would be present in a cohort of women with postnatal prolapse in whom the majority of prolapse would resolve within 12 months of birth [9].

To investigate this we performed a prospective cohort study comparing vaginal Aα and Aβ sensory nerve function in women with POP and without POP between 8 and 12 weeks and 6 months postnatally.

## Materials and Methods

### Study Design

One hundred and fifty primiparous women were recruited from the antenatal clinics at a tertiary hospital. Exclusion criteria were lack of capacity to consent, language barrier, previous pelvic floor surgery, female genital mutilation, medical comorbidities predisposing to sensory impairment, such as diabetes, and neuromodulatory medication.

Ethical approval for the study was obtained from the North West Greater Manchester West Research Ethics Committee (GMWest-14/NW/1316) and the Health Research Authority UK. Written informed consent was provided and all research was performed in accordance with relevant guidelines/regulations.

Testing was performed at 3 months (defined as between 8 and 12 weeks) and at 6 months postnatally (defined as between 20 and 24 weeks).

### Prolapse Examination

Pelvic examination for POP was performed using the internationally recognised Pelvic Organ Prolapse Quantification (POP-Q) system [14]. POP was defined a priori as the distal-most portion of the anterior or posterior vaginal wall at or below the level of the hymen, and the uterus at or below the lower third of the vagina (equivalent to POP-Q ≥ stage II) [15–17].

### Neurophysiology Methodology

Quantitative sensory testing was performed using the method of limits, whereby the stimulus is linearly increased until the woman indicates that she has perceived the sensation, called the perception threshold.

Prior to testing the woman received a standardised explanation of each procedure including a demonstration on her hand to familiarise her with the sensation. All testing was performed in one of three quiet examination rooms, with identical examination couches and the lithotomy stirrups at the same height for each assessment.

Women were blinded to perception-threshold readings to prevent repeatability bias. The researcher was blinded to the results of the first test when performing the second test. The 3 months between testing meant that the researcher was unable to recall the results of the first test when performing the second.

All sensory testing was performed using a standardised protocol to ensure the same location across the clinical research visits to prevent testing or repeatability bias.

#### Stretch Sensation (Aα Nerve Fibres)

Stretch sensation was used to test for Aα nerve fibres [12]. The ano-rectal catheter from Ardmore Healthcare was lubricated and inserted 4 cm into the vagina, the stimulus intensity, or volume of air, was linearly increased at a rate of 2 cm^3^ of air per second using a 60-ml syringe until the subject verbally indicated a sensation of stretching. The volume, or perception threshold, was recorded and the catheter deflated.

#### Vibration Sensation (Aβ Nerve Fibres)

Vibration sensation was used to test for Aβ nerve fibres [13]. A height-adjustable probe attached to a computer program was inserted 2–3 cm into the vagina. The computer program linearly increased the vibration amplitude until the woman indicated perception of the vibration using a response button held in her dominant hand. At this point the vibration stopped and the computer recorded the value, or perception threshold. This process was repeated six times and the average value calculated.

### Statistical Analysis

Pelvic Organ Prolapse Quantification stages were dichotomised into POP, defined as leading edge at or beyond the hymen, and no POP, defined as leading edge above the hymen. POP-Q measurements Ba, Bp and C were used to quantify the most dependent part of the anterior and posterior vaginal walls and cervix. There were no cases where Aa or Ap were less than Ba or Bp.

Vibration and stretch perception thresholds had a non-parametric distribution and data were analysed using Mann–Whitney *U* test for non-matched pairs, with Dunn’s test for pairwise comparisons.

This was an ancillary analysis and as such did not have a power calculation. A post hoc estimation used data from a pilot study that demonstrated that a difference in vibration sensation of 10% was clinically significant. In order to reject the null hypothesis (α = 0.05, β = 80) a minimum of 16 subjects was required per group to detect a 10% change.

## Results

One hundred and fifty women initially consented and underwent a POP-Q and baseline QST in the third trimester. There was no evidence of prolapse on POP-Q at the antenatal visit in any of the women.

Of these, 124 women attended postnatally for testing at 3 months (7 were uncontactable, 5 declined to attend, 4 gave birth elsewhere, 7 could not attend within the testing window and 1 emigrated), and 114 women re-attended at 6 months (4 were uncontactable, 3 could not attend within the testing window, 2 emigrated and 1 moved out of the region). Cohort demographics can be seen in Table 1.

**Table 1:** Cohort demographics

Demographics		Statistic
Age, years, median (IQR)		32.0 (29–34.5)
BMI, kg/m^2^, median (IQR)		24.2 (21.5–28.7)
Ethnicity, frequency (%)	White	36 (90.0)
	Asian	1 (2.5)
	Afro-Caribbean	3 (7.5)
	Oriental	0
Mode of delivery, frequency (%)	CS	36
	NVD	48
	AVD	40

*IQR* interquartile range, *CS* caesarean section, *NVD* normal vaginal delivery, *AVD* assisted vaginal delivery

There was no significant difference in age, BMI or ethnicity between women with and without POP at 3 or 6 months postnatally. All POP developed following normal and assisted vaginal births, with no cases of POP developing after caesarean birth.

There were no cases of uterine POP and all documented prolapse were within POP-Q stage II.

Anterior-compartment POP was present in 17.7% of women (22 out of 124), comprising 18.8% (9 out of 48) with normal vaginal delivery (NVD) and 32.5% (13 out of 40) with assisted vaginal delivery (AVD). There was no difference in the incidence of anterior-compartment POP between women with NVD and those with AVD (Chi-squared test, *p* = 0.138).

Posterior-compartment POP was present in 23.4% of all women (29 out of 114), 22.9% (11 out of 48) in the NVD group and 45.0% (18 out of 40) in the AVD group. Women were more likely to develop posterior-compartment POP following an AVD than after an NVD (Chi-squared test, *p* = 0.028).

**Fig. 1:**
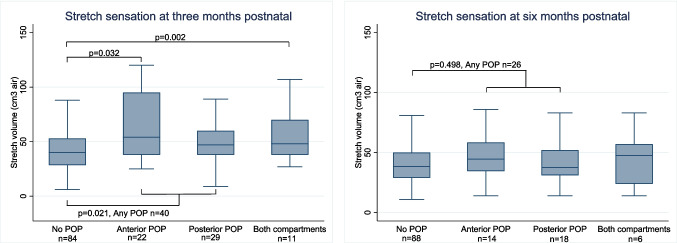
Stretch sensation in women with and without postnatal prolapse. Higher volumes produce a greater stretch of tissues and equate to reduced sensation. Data were analysed using Mann–Whitney *U* test, and groupwise comparisons were made using Dunn’s test; only significant probabilities are shown. *POP* pelvic organ prolapse (defined as the distal-most part of the vaginal wall at or below the level of the hymen); both compartments = anterior and posterior wall POP

At 3 months postnatally, women with POP demonstrated an impaired stretch sensation compared with women without POP (Fig. 1). By 6 months postnatally there was no difference in stretch sensation between women with POP and those without POP (Fig. 1).

**Fig. 2:**
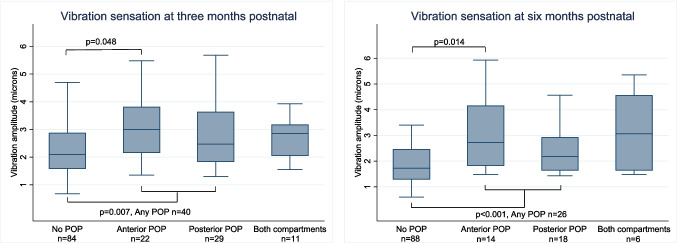
Vibration sensation in women with and without postnatal prolapse. Higher vibration amplitudes produce a stronger vibration and equate to reduced sensation. Data were analysed using Mann–Whitney *U* test, and groupwise comparisons were made using Dunn’s test; only significant probabilities are shown. *POP* pelvic organ prolapse (defined as the distal-most part of the vaginal wall at or below the level of the hymen); both compartments = anterior and posterior wall POP

There was a significant difference in vibration sensation at 3 months postnatally. Women with POP demonstrated a reduced vibration sensation compared with women with no prolapse (Fig. 2). At 6 months postnatally there remained a significant difference in vibration sensation in women with POP compared with women without POP (Fig. 2).

## Discussion

### Findings and Current Evidence

To our knowledge, this is the first study to evaluate Aα and Aβ sensory nerve function in women with and those without postnatal POP. The results show that the stretch sensation is reduced in women with POP at 3 months postnatally compared with women with no POP and recovers by 6 months postnatally. The vibration sensation was reduced in women with POP at both 3 and 6 months postnatally compared with women without POP. Our findings suggest that the sensory impairment seen in women with POP might occur at the time of childbirth, rather than part of the process of prolapse development that occurs later in life.

Women with prolapse in both compartments did not appear to display sensory impairment compared with women without prolapse. This likely represents a type II statistical error due to the small group sizes of 11 and 6. Visual inspection of Figs. 1 and 2 shows that the median perception thresholds for both compartments were higher than in the group of women without prolapse.

Two studies have evaluated vaginal sensation in women with prolapse. The first study by North et al. reported an abnormal vibration sensation in women with prolapse compared with age-matched normative data [7]. The study was limited by the small sample size and lack of comparison with controls. The second study by Gruenwald et al. described reduced temperature and vibration sensation in women with prolapse compared with age-matched controls [8]. They did not find any association between the severity of prolapse and degree of sensory impairment. Neither study performed an assessment of stretch sensation, as this methodology was described later [12].

Women with POP have evidence of motor nerve fibre denervation and reduced diameter of muscle fibres compared with controls [5, 18]. Research to date suggests that this motor denervation and muscle injury might occur during childbirth [6, 19, 20]. Earlier research by the authors found that vaginal sensation for both stretch and vibration deteriorated after vaginal birth [4]. Neural immunochemistry has also demonstrated reduced nerve fibres in women with POP [21–24].

The relative recovery seen in sensory-nerve function in women with postnatal POP in this study corresponds to the time to peripheral nerve reinnervation quoted as three to six months [25]. At the same time postnatal vaginal remodelling occurs, with around 80% of women with POP postnatally recovering by 12 months [9].

One explanation is that our findings represent the first measurable presentation of the sensory dysfunction seen in POP, and these are the women who will develop prolapse again later in life, after further mechanical pressure on their pelvic floor and age-related degeneration of nerves, muscles and connective tissues has occurred. Another explanation is that there are two types of prolapse, those related to childbirth injury that present early and those unrelated to childbirth injury that present later in life as the result of age-related pelvic floor changes.

### Strengths and Limitations

To our knowledge, this was the first study to perform sensory testing on women with and those without postnatal POP. Although this was an ancillary analysis, a post hoc power estimation confirmed that our sample size was more than adequate. Another strength of the study was the low attrition rate for a postnatal study. Women were recruited antenatally to the study and so it was not possible to predict who would develop prolapse postnatally. Thus, it was not possible to match the two groups for age or birth weight, although on post hoc analysis there was no significant difference in age between women with prolapse and those without. Earlier research by the authors reported no association between birth weight and postnatal pelvic sensation [4].

One limitation of the study is the inherent subjective nature of QST, although this was mitigated by strict adherence to the testing protocol.

This study did not include an assessment of Aδ and C nerve fibres using temperature sensation. A previous study in our unit had received complaints regarding the extended time frame needed to perform temperature testing and this led to concerns from the ethical committee regarding the research burden that this would place on postnatal women.

The 6-month postnatal follow-up period meant that the study did not evaluate what happened to pelvic sensation in the 80% of women whose postnatal prolapse would be expected to resolve by 12 months [9]. Ethical approval was granted to recruit women at any stage during pregnancy and continue in the study until 6 months postnatally, a total of 15 months in some cases. This ancillary analysis was not performed until data collection on the last woman had been completed, at which point the first woman was beyond 12 months postnatally and it was not possible to extend ethical approval to facilitate further testing at 12 months.

The study was unable to test women in the puerperium owing to ethical concerns surrounding vaginal sensation testing this soon after childbirth. Another limitation is the lack of somatosensory evoked potentials (SSEPs). SSEPs involve sending an electrical signal from genital to cephalic electrodes and measuring the response in the central nervous system.

### Generalisability

This study adds to the evidence base by furthering our understanding of the neuro-pathophysiology contributing to the development of POP. The pathophysiology of prolapse remains incompletely understood and this may be the reason for the high rate of prolapse recurrence after surgery. A better understanding of the pathophysiology of prolapse could help to develop more successful treatment options.

### Further Work

Further long-term observational research is needed to monitor the relationship between the sensory impairment seen in women with postnatal POP and developing POP in later life.

## Conclusion

In summary, this study demonstrates that the sensory impairment seen in women with POP occurs at the time of childbirth. Our findings add to the evidence on the pathophysiology of POP. Further work is needed to determine if this sensory impairment fully resolves as the anatomical POP resolves, or whether it persists and plays a role in the pathophysiology of POP in later life.

## Data Availability

The data that support the findings of this study are not openly available due to reasons of sensitivity and are available from the corresponding author upon reasonable request. Data are located in controlled access data storage at Manchester Foundation Trust NHS UK. Example from: 10.1186/s12910-022-00758-z.

## References

[1] Vos T, Flaxman A, Naghavi M, Al E. Years lived with disability (YLDs) for 1160 sequelae of 289 diseases and injuries 1990–2010: a systematic analysis for the Global Burden of Disease Study 2010. Lancet. 2012;380(9859):2163–96. 23245607 10.1016/S0140-6736(12)61729-2 PMC6350784

[2] Subak LL, Waetjen LE, Eeden SVD, Thom DH, Vittinghoff E, Brown JS. Cost of pelvic organ prolapse surgery in the United States. Obstet Gynecol. 2001;98(4):646–51. 11576582 10.1016/s0029-7844(01)01472-7

[3] Sung VW, Washington B, Raker CA. Costs of ambulatory care related to female pelvic floor disorders in the United States. Am J Obstet Gynecol. 2010;202(5):483.e1–4. 20227673 10.1016/j.ajog.2010.01.015 PMC2866792

[4] Mahoney CK, Reid FM, Smith ARB, Myers JE. The impact of pregnancy and childbirth on pelvic sensation: a prospective cohort study. Sci Rep. 2023;13(1):1535. 36707642 10.1038/s41598-023-28323-7 PMC9883213

[5] Smith ARB, Hosker GL, Warrell DW. The role of partial denervation of the pelvic floor in the aetiology of genitourinary prolapse and stress incontinence of urine. A neurophysiological study. Br J Obstet Gynaecol. 1989;96(9):24–8. 2923840 10.1111/j.1471-0528.1989.tb01571.x

[6] Allen RE, Hosker GL, Smith AR, Warrell DW. Pelvic floor damage and childbirth: a neurophysiological study. Br J Obstet Gynaecol. 1990;97(9):770–9. 2242361 10.1111/j.1471-0528.1990.tb02570.x

[7] North CE, Creighton SM, Smith A. A comparison of genital sensory and motor innervation in women with pelvic organ prolapse and normal controls including a pilot study on the effect of vaginal prolapse surgery on genital sensation: a prospective study. BJOG. 2013;120(2):193–9. 23240799 10.1111/1471-0528.12083

[8] Gruenwald I, Mustafa S, Gartman I, Lowenstein L. Genital sensation in women with pelvic organ prolapse. Int Urogynecol J. 2015;26(7):981–4. 25715930 10.1007/s00192-015-2637-5

[9] Hill AJ, Yang J, Martinez L, Nygaard I. Trajectories of pelvic floor symptoms and support following vaginal delivery in primiparas between third trimester and 1 year postpartum. Female Pelvic Med Reconstr Surg. 2021;27(8):507–13. 34397607 10.1097/SPV.0000000000001068 PMC9037832

[10] MacLennan AH, Taylor AW, Wilson DH, Wilson D. The prevalence of pelvic floor disorders and their relationship to gender, age, parity and mode of delivery. Br J Obstet Gynaecol. 2000;107(12):1460–70. 10.1111/j.1471-0528.2000.tb11669.x 11192101

[11] Fruhstorfer H, Lindblom U, Schmidt WC. Method for quantitative estimation of thermal thresholds in patients. J Neurol Neurosurg Psychiatry. 1976;39(11):1071–5. 188989 10.1136/jnnp.39.11.1071 PMC1083305

[12] Mahoney C, Chok SM, Bryant A, Myers J, Smith A, Reid F. Method of limits: female genital stretch perception thresholds. Neurourol Urodyn. 2020;39(2):778–84. 31961957 10.1002/nau.24282

[13] Vardi Y, Gruenwald I, Sprecher E, Gertman I, Yartnitsky D. Normative values for female genital sensation. Urology. 2000;56(6):1035–40. 11113756 10.1016/s0090-4295(00)00850-5

[14] Bump RC, Mattiasson A, Bø K, Brubaker LP, DeLancey JO, Klarskov P, et al. The standardization of terminology of female pelvic organ prolapse and pelvic floor dysfunction. Am J Obstet Gynecol. 1996;175(1):10–7. 8694033 10.1016/s0002-9378(96)70243-0

[15] Swift S, Woodman P, O’Boyle A, Kahn M, Valley M, Bland D, et al. Pelvic Organ Support Study (POSST): The distribution, clinical definition, and epidemiologic condition of pelvic organ support defects. Am J Obstet Gynecol. 2005;192(3):795–806. 15746674 10.1016/j.ajog.2004.10.602

[16] Bradley CS, Nygaard IE. Vaginal wall descensus and pelvic floor symptoms in older women. Obstet Gynecol. 2005;106(4):759–66. 16199633 10.1097/01.AOG.0000180183.03897.72

[17] Nygaard I, Brubaker L, Zyczynski H, Cundiff G, Richter H, Gantz M, et al. Long-term outcomes following abdominal sacrocolpopexy for pelvic organ prolapse. JAMA. 2013;309(19):2016–24. 23677313 10.1001/jama.2013.4919 PMC3747840

[18] Gilpin SA, Gosling JA, Smith ARB, Warrell DW. The pathogenesis of genitourinary prolapse and stress incontinence of urine. A histological and histochemical study. Br J Obstet Gynaecol. 1989;96(9):15–23. 2923839 10.1111/j.1471-0528.1989.tb01570.x

[19] Sultan AH, Kamm MA, Hudson CN. Pudendal nerve damage during labour: prospective study before and after childbirth. Br J Obstet Gynaecol. 1994;101(1):22–8. 8297863 10.1111/j.1471-0528.1994.tb13005.x

[20] Snooks SJ, Swash M. Abnormalities of the innervation of the urethral striated sphincter musculature in incontinence. Br J Urol. 1984;56(4):401–5. 6335972 10.1111/j.1464-410x.1984.tb05830.x

[21] Busacchi P, Perri T, Paradisi R, Oliverio C, Santini D, Guerrini S, et al. Abnormalities of somatic peptide-containing nerves supplying the pelvic floor of women with genitourinary prolapse and stress urinary incontinence. Urology. 2004;63(3):591–5. 15028474 10.1016/j.urology.2003.09.017

[22] Hoyle CH, Stones RW, Robson T, Whitley K, Burnstock G. Innervation of vasculature and microvasculature of the human vagina by NOS and neuropeptide-containing nerves. J Anat. 1996;188:633–44. 8763480 PMC1167491

[23] Inal H, Kaplan P, Usta U, Tastekin E, Aybatl A, Tokuc B. Neuromuscular morphometry of the vaginal wall in women with anterior vaginal wall prolapse. Neurourol Urodyn. 2010;29(3):458–63. 19714736 10.1002/nau.20779

[24] Kaplan P, Usta U, Inal H, Tastekin T, Tokuc B. Neuromuscular morphometry of uterine ligaments and vaginal wall in women with pelvic organ prolapse. Neurourol Urodyn. 2011;30:126–32. 21046656 10.1002/nau.20972

[25] Menorca RMG, Fussell TS, Elfar JC. Peripheral nerve trauma: mechanisms of injury and recovery. Hand Clin. 2013;29(3):317–30. 23895713 10.1016/j.hcl.2013.04.002 PMC4408553

